# Giant Colloid Cyst: A Rare Etiology for Chronic Headache

**DOI:** 10.7759/cureus.36876

**Published:** 2023-03-29

**Authors:** Ahmed Y Bukannan, Mahdi Aljawad

**Affiliations:** 1 Radiology, Salmaniya Medical Complex, Manama, BHR; 2 Radiology, Qatif Central Hospital, Qatif, SAU

**Keywords:** case report, magnetic resonance imaging, colloid cyst, papilledema, syncope, chronic headache

## Abstract

Chronic headache affects a significant proportion of the population and can be caused by an underlying lesion or a serious condition. This case report describes a 38-year-old male with a history of chronic migraine headaches who presented with syncope. The patient was found to have bilateral papilledema on fundoscopic examination and a well-circumscribed, oval-shaped lesion located within the intraventricular septum on MRI. The lesion was identified as a colloid cyst and was surgically removed through endoscopic transnasal excision. The patient’s symptoms improved significantly postoperatively, including the resolution of his chronic headaches and syncope. This case report highlights the importance of considering space-occupying lesions as a possible cause of chronic headaches, particularly when symptoms do not respond to conventional treatments. It demonstrates that an endoscopic transnasal resection is a feasible approach, even for large colloid cysts.

## Introduction

Chronic headache is a neurological condition that affects a significant proportion of the population and can be a manifestation of an underlying lesion or a more serious condition. The prevalence of chronic headaches is high, with estimates suggesting that up to 4% of the global population suffers from this condition [1]. Despite its high prevalence, the etiology of chronic headaches remains poorly understood, and it can be challenging to diagnose and manage. In some cases, chronic headaches may be due to an underlying lesion. A colloid cyst is a relatively rare type of benign brain tumor that can cause significant neurological symptoms. These cysts are composed of a gelatinous material known as colloid and typically occur in the third ventricle of the brain [2]. Although colloid cysts are usually small and benign, they can grow to large sizes and cause life-threatening complications, such as obstructive hydrocephalus. In this case report, we present a rare case of a giant colloid cyst in a patient with a chronic headache, highlighting the potential severity of this condition and the importance of early diagnosis and management [1-2].

## Case presentation

We present a case of a 38-year-old male patient with a history of chronic headaches for the past two years, initially diagnosed as a migraine headache due to its diffuse nature, moderate to severe intensity, and daily occurrence. Despite using over-the-counter analgesics, the patient did not experience significant relief, which negatively affected his quality of life, causing him to miss work and social activities.

On the day of the presentation, the patient experienced a new symptom of syncope while sitting at his desk at work. The patient reported feeling dizzy and nauseous before losing consciousness for a few seconds. The syncope episode was an alarming development, and he was immediately taken to the emergency department for evaluation.

The patient’s past medical history was remarkable for hypertension for the past five years, managed with captopril 25 mg, and diabetes mellitus for the past seven years, managed with metformin 500 mg twice daily. The patient also had a significant family history of migraine in his mother and sister. He was a smoker, with a 20-year history of smoking one pack of cigarettes per day, and denied any alcohol consumption.

During the physical examination, the patient was alert and oriented and showed no signs of neurological deficits. Vital signs were within normal limits, including a blood pressure of 122/76 mmHg, a heart rate of 78 beats per minute, a respiratory rate of 16 breaths per minute, and a temperature of 98.6°F. The neurological examination did not reveal any signs of neurological deficit. However, a fundoscopic examination revealed bilateral papilledema. A complete blood count and electrolyte panel both showed normal results, and liver function tests were also within normal limits (Table 1).

**Table 1:** Summary of laboratory investigations

Lab parameters	Result	Reference range
White blood cell count	7.2 x 10^9^/L	4.0-11.0 × 10^9^/L
Hemoglobin	14.2 g/dL	12.0-15.5 g/dL
Platelet count	235 x 10^9^/L	150-450 × 10^9^/L
Sodium	139 mEq/L	135-145 mEq/L
Potassium	4.0 mEq/L	3.5-5.0 mEq/L
Chloride	100 mEq/L	98-107 mEq/L
Alanine aminotransferase	25 U/L	0-40 U/L
Aspartate aminotransferase	31 U/L	0-35 U/L
Alkaline phosphatase	84 U/L	35-104 U/L

**Figure 1:**
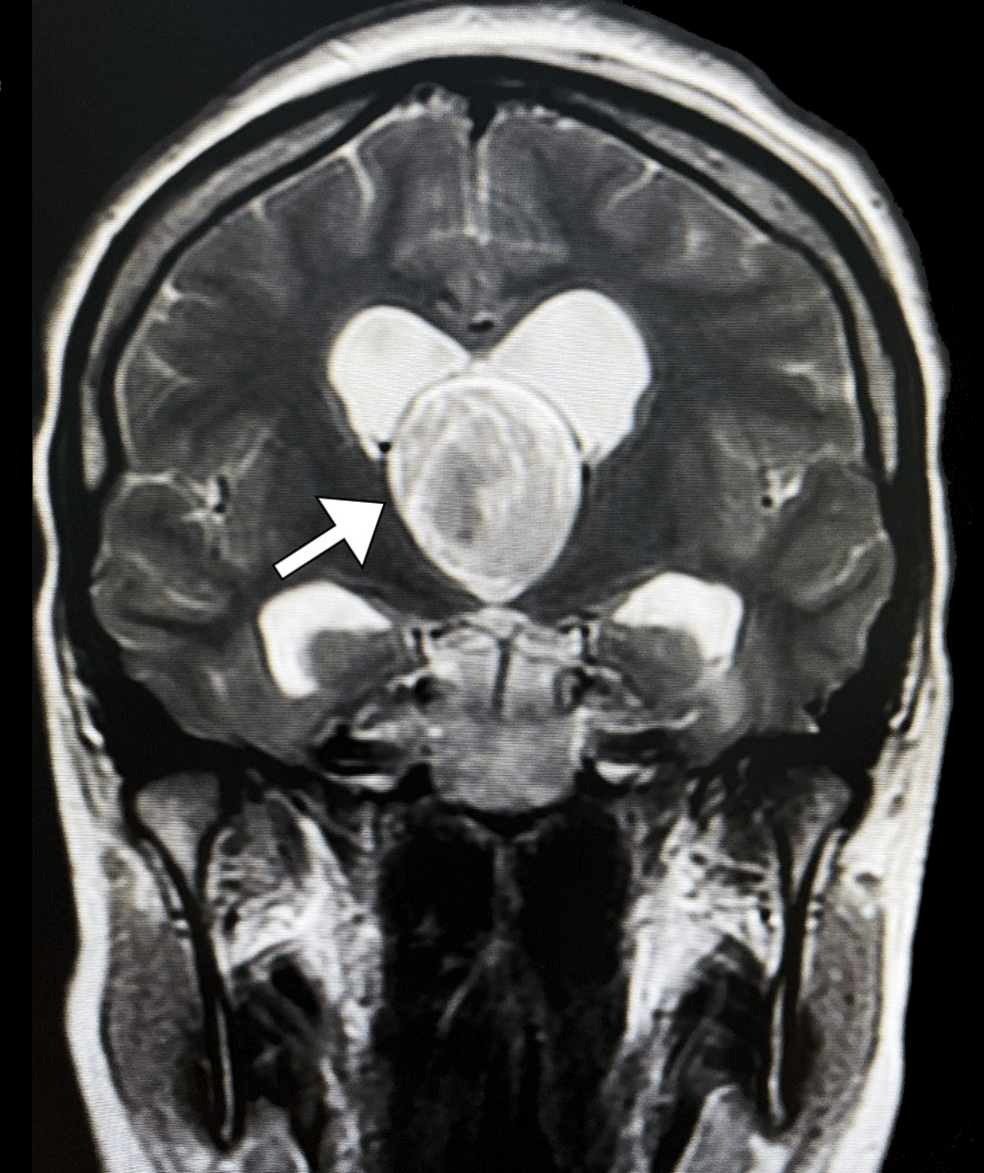
Coronal T2-weighted MRI image of the brain showing a large, heterogeneous mass arising from the roof of the third ventricle, resulting in obstructive hydrocephalus

**Figure 2:**
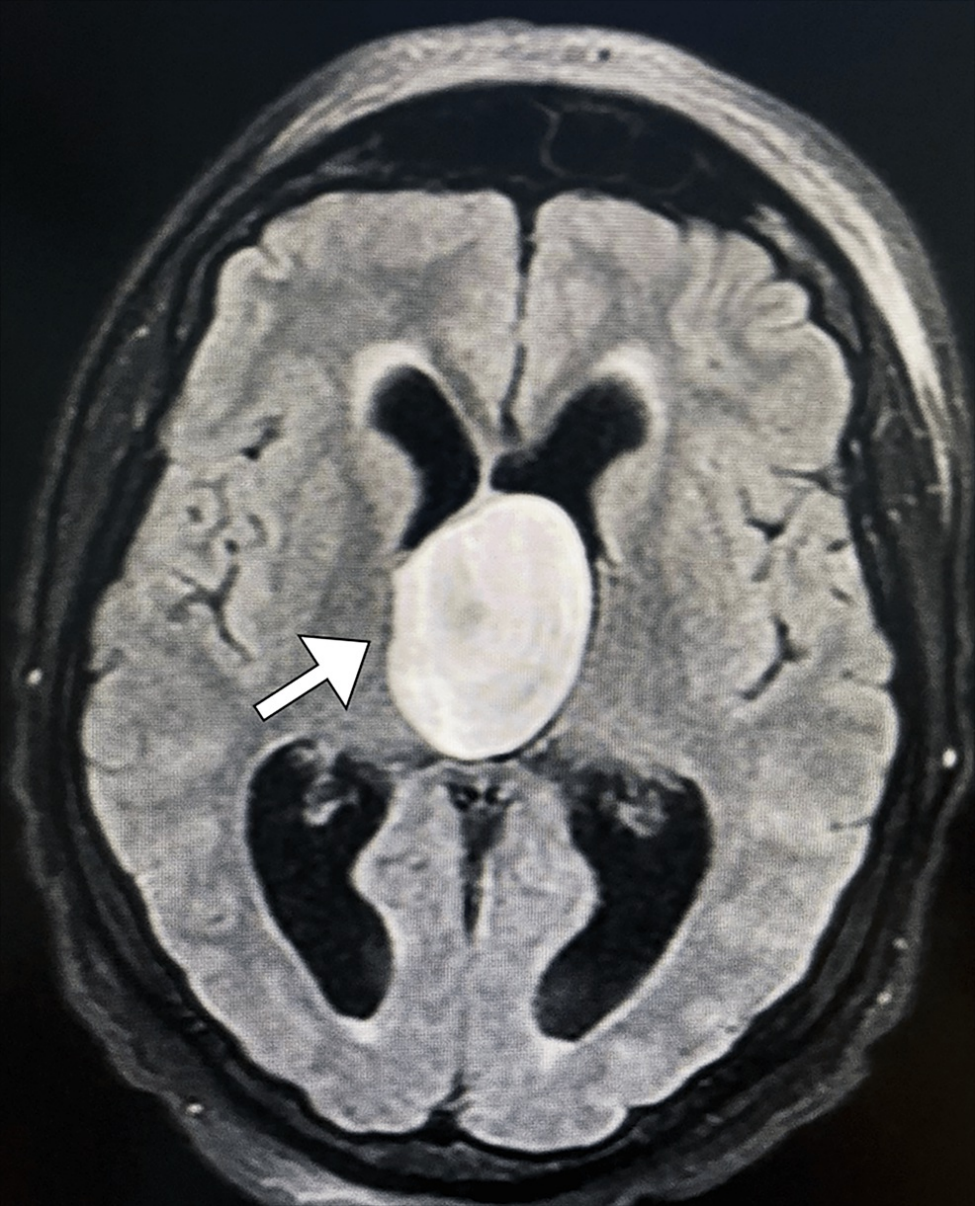
Axial FLAIR MRI image of the brain displaying a large, heterogeneous mass arising from the roof of the third ventricle, resulting in obstructive hydrocephalus FLAIR: fluid-attenuated inversion recovery

After further diagnostic workup, MIR of the brain revealed a well-circumscribed oval-shaped lesion measuring 3.9 cm x 6.1 cm x 4.8 cm at its maximum dimensions, located within the intraventricular septum adjacent to the foramen of Monro. The lesion had high signal intensity on T1-weighted images and heterogeneous signal intensity on T2-weighted images. Additionally, it was associated with significant dilatation of both lateral ventricles and periventricular increased signal intensity, representing transependymal edema (Figures 1-2).

The patient underwent urgent surgical intervention with endoscopic transnasal excision of the colloid cyst. During the hospital course, the patient was closely monitored for any complications related to the surgery. He received post-operative care, which included analgesics, prophylactic antibiotics, and fluid management. The patient’s symptoms improved significantly postoperatively, and he reported complete resolution of his chronic headaches, and there were no further episodes of syncope.

## Discussion

Chronic headache is a common presenting symptom in neurology clinics and emergency departments. The majority of these headaches are due to benign conditions, such as tension-type headaches or migraines, and can be managed with over-the-counter analgesics or preventive medications [1]. However, certain red flags suggest a more serious underlying pathology, such as a colloid cyst. Our patient’s chronic headaches were initially diagnosed as migraines, highlighting the importance of considering intracranial pathology in patients with chronic headaches, especially when symptoms are not responding to conventional treatments. A comprehensive history and physical examination are the initial steps in evaluating patients with chronic headaches [1]. However, these examinations may not always reveal the underlying pathology, and further diagnostic workup may be required. Neuroimaging, such as CT, is often necessary to identify structural abnormalities.

Colloid cysts are typically slow-growing tumors that arise from remnants of the primitive neuroepithelium. They are usually found incidentally in neuroimaging studies or may present with nonspecific symptoms such as headache, dizziness, nausea, vomiting, or visual changes [2]. Rarely, they may present with acute neurological symptoms such as seizures, sudden onset of severe headaches, or syncope [3]. The pathophysiology of colloid cysts is not well understood, but they are thought to arise from embryonic remnants of the primitive neuroepithelium. Colloid cysts are filled with a thick, viscous fluid that is rich in protein and cholesterol, and they are lined by a layer of the columnar or cuboidal epithelium [4]. The mechanism by which these cysts cause symptoms is related to the obstruction of the third ventricle, leading to increased intracranial pressure and hydrocephalus [2].

In neuroimaging studies, colloid cysts are typically well-circumscribed, rounded, and hypodense lesions that enhance with contrast [4]. They are typically located in the third ventricle and can cause hydrocephalus by obstructing the foramen of Monro, which is the opening that connects the third ventricle to the lateral ventricles [2]. The size of colloid cysts varies widely, ranging from a few millimeters to several centimeters in diameter. The management of colloid cysts depends on their size, location, and symptoms.

Surgical intervention is typically indicated for colloid cysts that are causing symptoms or are larger than 1 cm in diameter [2,5]. The preferred surgical approach is endoscopic transnasal resection, which involves passing an endoscope through the nose and sinuses to access the third ventricle and remove the cyst [5]. This approach is less invasive than traditional open craniotomy and has a lower rate of complications. However, it requires specialized training and expertise and may not be feasible in all cases. In addition to surgical resection, some patients with colloid cysts may benefit from ventriculoperitoneal shunt placement. This procedure involves the placement of a catheter that drains cerebrospinal fluid from the ventricles to the peritoneal cavity, thereby relieving the pressure caused by the cyst. However, shunt placement is associated with a higher risk of complications, including infection, shunt malfunction, and overdrainage [3,5]. In addition, due to its minimal invasiveness and high effectiveness, the endoscopic removal of colloid cysts has gained widespread acceptance, leading to lower morbidity rates in comparison to microsurgical resection [6].

## Conclusions

This case report highlights the importance of considering space-occupying lesions as a possible cause of chronic headaches, particularly when symptoms do not respond to conventional treatments. Colloid cysts should be included in the differential diagnosis of chronic headaches, and early diagnosis and treatment are essential to prevent complications such as hydrocephalus and intracranial hypertension. The choice of surgical approach depends on several factors, including the size, location, and symptoms of the cyst. The case demonstrates that an endoscopic transnasal resection is a feasible approach, even for large colloid cysts.
